# Pull the fuzes: Processing protein precursors to generate apoplastic danger signals for triggering plant immunity

**DOI:** 10.1016/j.xplc.2024.100931

**Published:** 2024-04-30

**Authors:** Daniele Del Corpo, Daniele Coculo, Marco Greco, Giulia De Lorenzo, Vincenzo Lionetti

**Affiliations:** Department of Biology and Biotechnology “Charles Darwin,” Sapienza University of Rome, Rome, Italy

**Keywords:** apoplast, immunity activation, post-translational processing, cell wall, proteases, pro-peptides, pro-enzymes, phytocytokines, cell-wall remodeling enzymes, phytopathogenic microbes, plant immunity

## Abstract

The apoplast is one of the first cellular compartments outside the plasma membrane encountered by phytopathogenic microbes in the early stages of plant tissue invasion. Plants have developed sophisticated surveillance mechanisms to sense danger events at the cell surface and promptly activate immunity. However, a fine tuning of the activation of immune pathways is necessary to mount a robust and effective defense response. Several endogenous proteins and enzymes are synthesized as inactive precursors, and their post-translational processing has emerged as a critical mechanism for triggering alarms in the apoplast. In this review, we focus on the precursors of phytocytokines, cell wall remodeling enzymes, and proteases. The physiological events that convert inactive precursors into immunomodulatory active peptides or enzymes are described. This review also explores the functional synergies among phytocytokines, cell wall damage-associated molecular patterns, and remodeling, highlighting their roles in boosting extracellular immunity and reinforcing defenses against pests.

## Teaser

The apoplast is a crucial cellular compartment for the control of phytopathogenic microbes. Plants use complex surveillance mechanisms to detect surface threats and activate immunity. This process requires precise regulation to effectively trigger defense responses, which involve the activation of proteins and enzymes through post-translational processing. This review delves into the activation of precursors of phytocytokines, cell-wall enzymes, and proteases, highlighting their roles in enhancing extracellular immunity against pests.

## Introduction

Plants are sessile organisms constantly threatened by biotic stresses. Pathogens employ diverse infection strategies to obtain nutrients from plants, leading to diseases. They can be categorized on the basis of their different lifestyles (McCombe et al., 2022). Necrotrophs kill host cells to access nutrients, biotrophs feed on living plants by weakening the plant immune system, and hemibiotrophs initially extract nutrients from living tissues before switching to a necrotrophic phase. The apoplast, i.e., the intercellular space beyond the plasma membrane, serves as an active battlefield between plants and invading microbes (Sattelmacher 2001; Wang et al., 2020; Dora et al., 2022). Several events that occur in this compartment contribute to efficiently countering dangerous microbes (Dora et al., 2022; Vicré and Lionetti 2023). Unlike animals, plants lack an adaptive somatic immune system and rely solely on their innate cellular immune system, which is responsible for surveilling and perceiving pathogenic microbes and rapidly activating appropriate defenses (Rui and Dinneny 2020; DeFalco and Zipfel 2021; Ngou et al., 2022). Plants monitor the presence of danger signals in the apoplast environment via cell-surface-localized pattern-recognition receptors (PRRs), categorized as receptor kinases (RKs) or receptor proteins (Zipfel 2014; Saijo et al., 2018). These sensors can detect foreign conserved molecules derived from microbes, nematodes, insects, and parasitic plants, known as pathogen- or microbe-associated molecular patterns (PAMPs or MAMPs), thereby activating pattern-triggered immunity (PTI) (Ge et al., 2022).

**Figure 1:**
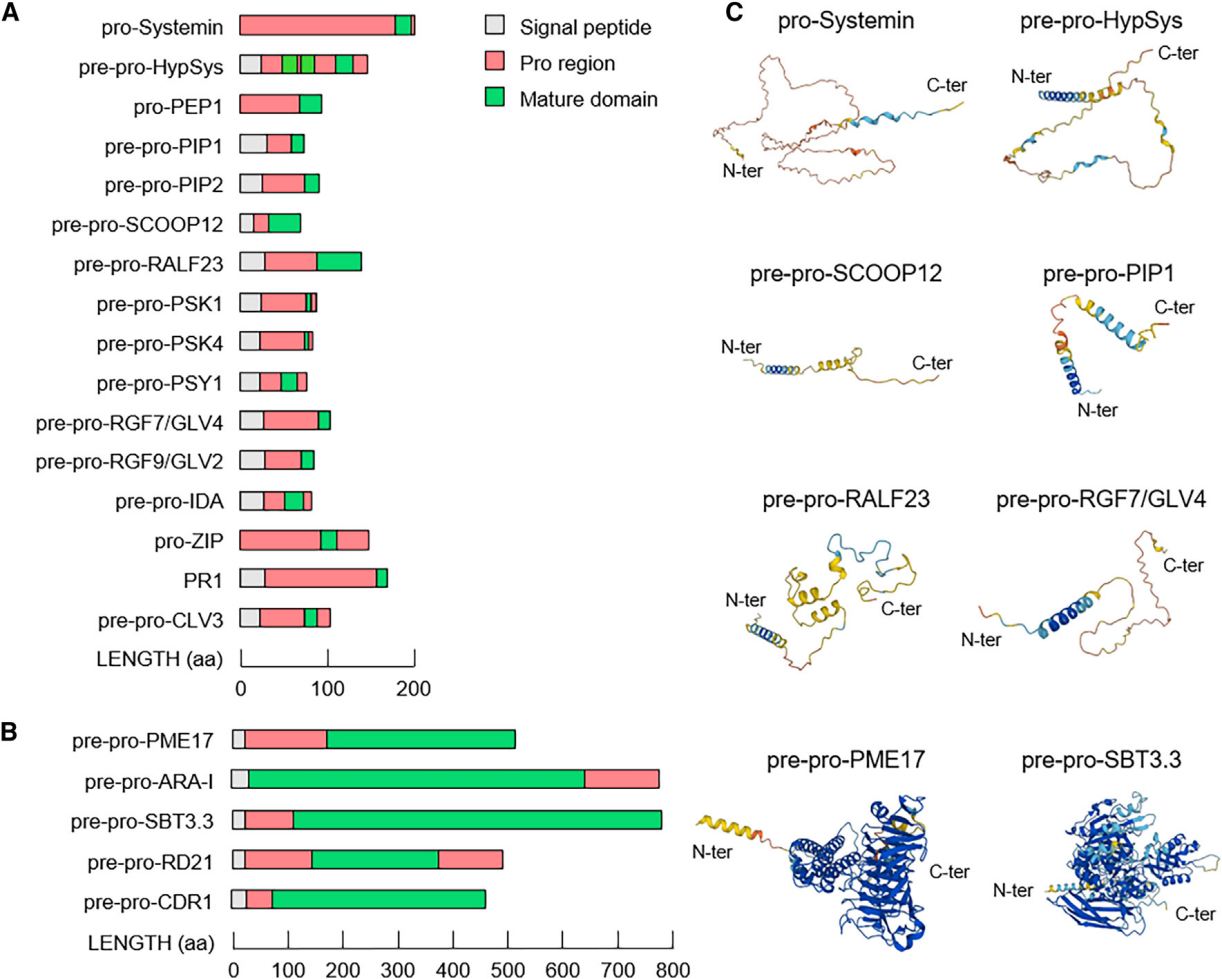
Multidomain organization of protein precursors with immunomodulatory functions. Schematic representation of the modular structures of the main **(A)** pro-peptides and **(B)** pro-enzymes discussed in the review. The lengths and positions of domains in the precursors are scaled using information obtained from UniProt (https://www.uniprot.org/). The N-terminal signal peptide (gray box), pro region (pink box), and active domain (green box) are indicated. The scales below indicate the domain lengths expressed in number of aa. **(C)** AlphaFold (https://alphafold.ebi.ac.uk/) models of six representative phytocytokine precursors: pro-Systemin (UniProt accession P27058), pre-pro-HypSys (UniProt accession Q7XAD0), pre-pro-SCOOP12 (UniProt accession B3H7I1), pre-pro-PIP1 (UniProt accession Q1PE40), pre-pro-RALF23 (UniProt accession Q9LUS7), and pre-pro-RGF7/GLV4 (UniProt accession Q6NNL3); and two representative enzyme precursors: pre-pro-PME17 (UniProt accession O22149) and pre-pro-SBT3.3 (UniProt accession Q9MAP5). The AlphaFold models are shaded on the basis of the per-residue model confidence (pLDDT) score: dark blue represents the most confidently predicted regions, transitioning through light blue and yellow to orange for regions with very low confidence. N-ter, N-terminal; C-ter, C-terminal.

**Figure 2:**
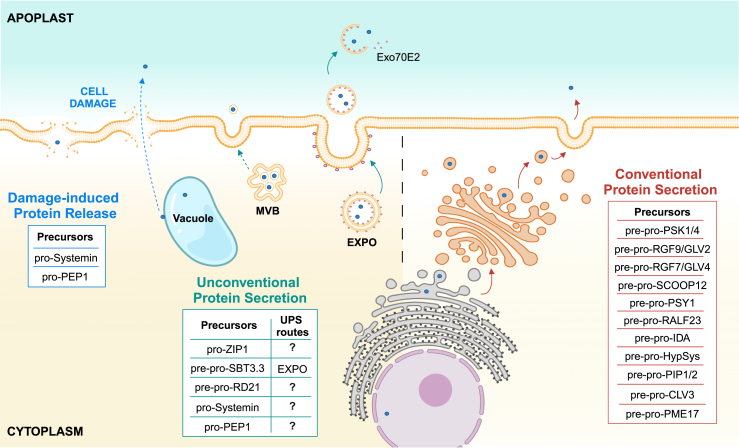
Schematic representation of putative secretion pathways of pro-peptides and pro-enzymes. Some precursors follow the conventional protein secretion pathway: the pro-enzyme/peptides are translocated into the ER, transported to the Golgi, and subsequently secreted into the extracellular space after fusion of the secretory vesicles with the plasma membrane. Other precursors can bypass the Golgi and follow the unconventional protein secretion pathway (left panel) to reach the apoplast. Exocyst-positive organelles (EXPOs) and multivesicular bodies (MVBs) appear to be particularly involved in this transport. Some precursors can be released in the apoplast after cell damage. Red, green, and blue arrows represent conventional protein secretion, unconventional protein secretion, and damage-induced protein release, respectively. Dashed arrows indicate paths that lack direct evidence. Blue dots represent peptide or protease precursors.

**Figure 3:**
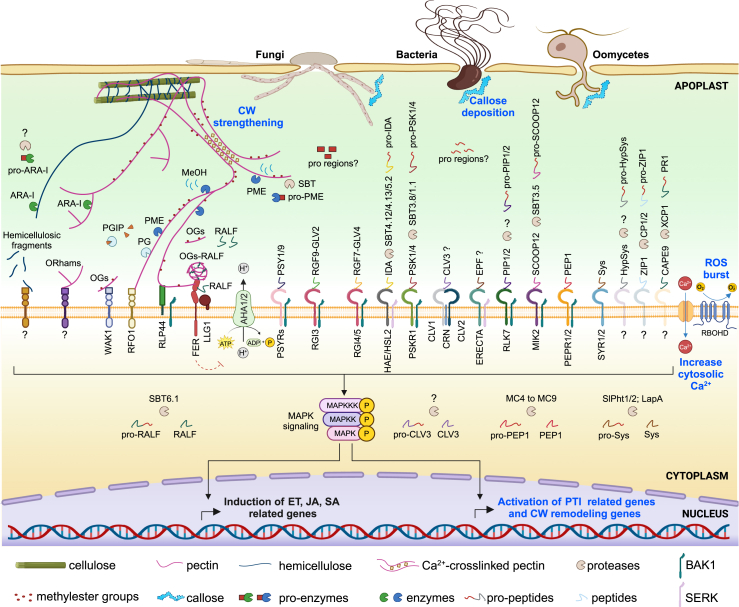
Overview of precursor maturation, perception, and function in plant immunity. During various pathogen infections, pro-proteins are processed by proteases and released into the apoplast, where they are recognized by specific cell surface sensors, resulting in the activation of plant defense responses. MAPKs may phosphorylate transcription factors (TFs), which control the expression of PTI-related genes (in blue are the typical PTI responses: ROS burst, increased cytosolic Ca^2+^ influx, callose deposition, and CW strengthening) as well as SA-, JA-, and/or ethylene (ET)-responsive genes that regulate immunity. The precursor forms of the CW remodeling enzymes (pro-PME and pro-ARA-I) can be processed to favor the production of oligosaccharides, which are elicitors of plant immunity. Oligogalacturonides (OGs) can be released by the combined action of polygalacturonases (PGs), polygalacturonase-inhibiting proteins (PGIPs), and PMEs. Processed PMEs can also perform pectin de-methylesterification. WAK1, RFO1, RLP44, and FERONIA can interact with de-methylesterified pectin. WAK1 senses OGs and FERONIA can sense condensed OG-RALFs to modulate plant defense responses. Oligorhamnogalacturonides (ORhams) can potentially be released by debranching enzymes such as the bifunctional α-L-arabinofuranosidase/β-D-xylosidases (ARA-I). The precursor/protease pairs and the putative or demonstrated subcellular compartment of maturation are shown. Green bars and pink and blue lines represent cellulose, pectin, and hemicellulose, respectively. Pink dots and diamond symbols represent methylester groups and Ca^2+^, respectively. Dashed arrows indicate pathways that lack direct evidence. RBOHD, respiratory burst oxidase homolog protein D; AHA1/2, Arabidopsis H^+^-ATPase isoforms 1 and 2.

Importantly, danger signals may also originate from immunogenic plant host factors (Gust et al., 2017). Indeed, PRRs can recognize plant small secreted peptides, referred to as phytocytokines (Figures 1, 2, and 3; Table 1) (Luo 2012; Hou et al., 2021a; Tanaka and Heil 2021; Rzemieniewski and Stegmann 2022). These molecules are considered to be hormone-like compounds capable of acting locally and systemically in plant development and stress responses (Bartels and Boller 2015; Pastor-Fernández et al., 2023). Furthermore, PRRs can perceive other endogenous danger molecules, referred to as damage-associated molecular patterns (DAMPs), which activate a DAMP-triggered immunity that shares many features with PTI (Figures 1 and 3; Table 1). These elicitors encompass cytosolic proteins, peptides, nucleotides, and amino acids, potentially released during microbial infections or upon mechanical damage (Hou et al., 2019; Tanaka and Heil 2021). Several DAMPs originate from degradation of the plant cell wall (CW), a significant constituent of the apoplast (De Lorenzo and Cervone 2022; Martín-Dacal et al., 2023). The CW consists primarily of polysaccharides, comprising a complex mixture of cellulose, hemicelluloses, and pectins, alongside phenolic compounds, structural and enzymatically active proteins, ions, and water (Swaminathan et al., 2022). Pectin, a crucial source of CW-derived DAMPs, is composed of galacturonic acid–rich polysaccharides, including homogalacturonan (HG), rhamnogalacturonan I (RGI), and the substituted galacturonans rhamnogalacturonan II (RG-II) and xylogalacturonan (Mohnen 2008). Among the most well-characterized CW-derived DAMPs are oligogalacturonides (OGs), fragments of HG released by the combined action of microbial pectin hydrolytic enzymes such as polygalacturonases (PGs) and plant polygalacturonase-inhibiting proteins (Xiao et al., 2024). Loss of CW integrity induced by microbial enzymatic degradation can trigger various defense responses, including CW remodeling aimed at reinforcing the structure to maintain CW integrity and protect against pathogens (Bellincampi et al., 2014; Rui and Dinneny 2020). Pectin methylesterases (PMEs) and their inhibitors (PMEIs) can play significant roles in pectin remodeling and CW integrity signaling (Bethke et al., 2014; Lionetti et al., 2017).

**Table 1:** List of apoplastic protein precursors discussed in the manuscript, with their domain lengths, their cognate proteases, and the subcellular compartments where their processing occurs.

Symbol	Full length (aa)	Pro region (aa)	Mature domain (aa)	Protease involved in cleavage	Processing compartment	References
pro-Systemin (LOC543989)	200	178; 4	18	SlPhyt1; SlPhyt 2; LapA		Pearce et al., 1991; Gu and Walling 2000; Beloshistov et al., 2018
pre-pro-HypSys (LOC543883)	146	24; 4; 25; 16	18; 15; 15		apoplast	Pearce and Ryan 2003; Chen et al., 2008
pro-PEP1 (AT5G64900)	92	69	23	MC4 to MC9	intracellular	Huffaker et al., 2006; Shen et al., 2019
pre-pro-PIP1 (AT4G28460)	72	29	13		apoplast	Miyashita et al., 2011; Hou et al., 2014
pre-pro-PIP2 (AT4G37290)	84	45	13		apoplast	Hou et al., 2014; Vie et al., 2015; Hussain et al., 2021
pre-pro-SCOOP12 (AT5G44585)	78	19	35	SBT3.5	apoplast	Hou et al., 2021b; Yang et al., 2023
pre-pro-RALF23 (AT3G16570)	138	60	50	SBT6.1		Srivastava et al., 2009; Xiao et al., 2019
pre-pro-PSK1 (AT1G13590)	87	52; 6	5	SBT3.8		Matsubayashi et al., 2006; Amano et al., 2007; Stührwohldt et al., 2021
pre-pro-PSK4 (AT3G49780)	79	49; 4	5	SBT1.1		Matsubayashi et al., 2006; Amano et al., 2007; Srivastava et al., 2008
pre-pro-PSY1 (AT5G58650)	75	25; 10	18			Amano et al., 2007; Matsubayashi 2011
pre-pro-RGF9/GLV2 (AT5G64770)	78	39	13			Matsuzaki et al., 2010; Ghorbani et al., 2016; Kaufmann and Sauter 2019
pre-pro-RGF7/GLV4 (AT3G02240)	102	62	13			Matsuzaki et al., 2010
pre-pro-IDA (AT1G68765)	77	23; 8	14	SBT4.12; SBT 4.13; SBT 5.2		Stenvik et al., 2008; Schardon et al., 2016; Stührwohldt et al., 2017
pro-ZIP1 (AC210027.3_FGP003)	137	87; 33	17	CP1; CP2	apoplast	Ziemann et al., 2018
PR1 (AT2G14610) (peptideCAPE9)	161	124	11	XCP1		Chien et al., 2015; Chen et al., 2023b
pre-pro-CLV3 (AT2G27250)	96	48; 14	12 or 13		intracellular	Rojo et al., 2002; Xu et al., 2013; De Marchis et al., 2018
pre-pro-PME17 (AT2G45220)	511	148	307	SBT3.5		Senechal et al., 2014; Del Corpo et al., 2020
pre-pro-ARA-I (AY029259)	777	130	615			Lee et al., 2003
pre-pro-SBT3.3 (AT1G32960)	777	87	666	self-activation		Ramírez et al., 2013; Coculo et al., 2023
pre-pro-RD21 (AT1G47128)	462	115; 110	216			Gu et al., 2012
pre-pro-CDR1 (AT5G33340)	437	48	364			Xia et al., 2004; Simões et al., 2007

The indicated full aa lengths of the protein domains were predicted by UniProt (https://www.uniprot.org/). Sequences that do not belong to the mature domain are considered to be pro regions.

A multitude of signaling proteins, along with their post-translational modifications, have emerged as strategic factors for alerting the plant to danger and facilitating a more effective and rapid immune response (Olsson et al., 2019; Stührwohldt and Schaller 2019). Indeed, numerous phytocytokines and enzymes involved in CW remodeling are initially synthesized as inactive precursors that can be processed by specific proteases to release the pro region from the biologically active domain (Figures 1 and 3; Table 1) (Matsubayashi 2014; Olsson et al., 2019). Their post-translational processing and modification serve as a timely and localized strategy for effective activation of plant immunity, CW reinforcement, and production and/or perception of DAMPs (Chen et al., 2020; Del Corpo et al., 2020; Stührwohldt et al., 2020; Coculo et al., 2023). Proteolytic events that activate certain immunogenic protein precursors can occur within minutes, producing rapid signals to alert the plant to imminent danger (Hander et al., 2019). Proteases can thus function as molecular switches, processing protein and peptide precursors involved in plant immunity (Table 1) (Wang et al., 2020; Godson and van der Hoorn 2021). Remarkably, many proteases are synthesized as precursors themselves, requiring maturation to release their activity (Figure 1B).

Protein precursors undergo processing in specific subcellular compartments, with the active portion targeted to the apoplast following conventional protein secretion or unconventional protein secretion (UPS) pathways (Wang et al., 2017a) (Figure 2). In conventional protein secretion, proteins follow the route of the endoplasmic reticulum–Golgi apparatus and the subsequent endomembrane system. By contrast, multiple UPS pathways have been proposed, including exocyst-positive organelles, multivesicular bodies, and vacuole-plasma membrane fusion (Ding et al., 2014). Interestingly, UPS is largely associated with plant defense responses, akin to mechanisms observed in animals (Maricchiolo et al., 2022).

Upon maturation and perception by PRRs, DAMPs and phytocytokines can trigger multiple immune responses aimed at restricting pathogen invasion (Figure 3) (De Lorenzo et al., 2019; Hou et al., 2021a). These include a transient reactive oxygen species (ROS) burst in the apoplast, an increase in cytosolic calcium (Ca^2+^) concentration, phosphorylation of receptor-like cytoplasmic kinases, activation of mitogen-activated protein kinases (MAPKs) and Ca^2+^-dependent protein kinases, transcriptional reprogramming, and expression of defense genes, as well as production of antimicrobial molecules (Ferrari et al., 2013; Zhou and Zhang 2020; DeFalco and Zipfel 2021; Ge et al., 2022). These induced defenses can be modulated by immune hormone crosstalk in which jasmonic acid (JA), salicylic acid (SA), and ethylene play dominant roles (Aerts et al., 2021).

**Figure 4:**
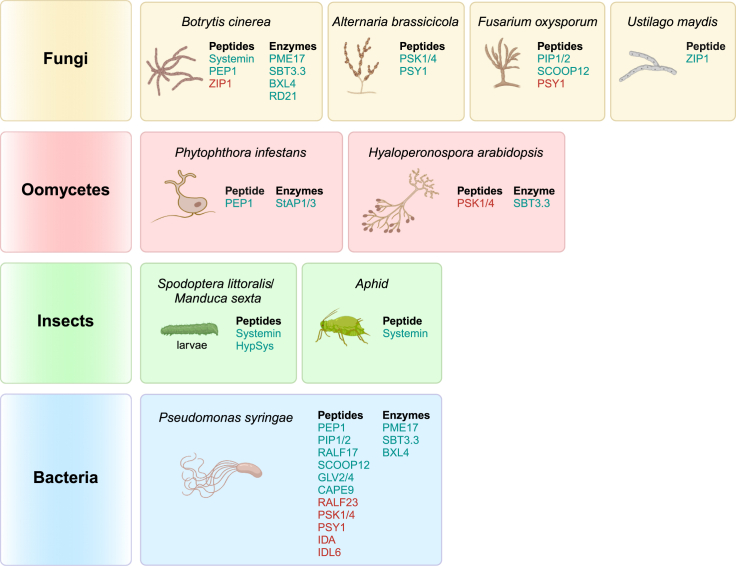
Schematic representation of the involvement of precursors in plant–pathogen interactions. Various plant pro-peptides and pro-enzymes can be generated and activated in response to specific pathogens. A single precursor can exhibit a wide range of effects, making it effective against multiple pathogens. Active peptides/enzymes that improve plant resistance are depicted in turquoise, and those that increase susceptibility are shown in red. All peptides/enzymes mentioned belong to *A. thaliana* except for Systemin, HypSys, ZIP1, and StAP1/3, which belong to *S. lycopersicum*, *Z*. *mays*, and *S*. *tuberosum*, respectively.

This review highlights the importance of promptly activating inactive precursors of immunogenic peptides, CW remodeling enzymes, and proteases as a clever strategy by which to enhance the effectiveness of alarm signals in plant immune responses. We provide a comprehensive overview of the most studied and characterized protein precursors, focusing on their predicted structures, regulation, maturation processes, and functions in plant immunity (Figures 1 and 3; Table 1). Some precursors exhibit broad-spectrum actions, whereas others contribute to specific resistance against pathogens with a particular lifestyle; others may also act as susceptibility factors (Figure 4). Furthermore, we outline open questions and propose directions for future research aimed at advancing our understanding of this topic.

## Processing of phytocytokine precursors is required for apoplastic immunity

The term “plant cytokines” or “phytocytokines” was coined to refer to small secreted peptides in plants that regulate both plant immunity and development, analogous to metazoan cytokines (Luo 2012). Several genes encoding phytocytokines are rapidly and significantly induced during pathogen infection or upon exogenous treatments with PAMPs, DAMPs, and other phytocytokines (Combest et al., 2021). These immunostimulatory peptides have recently been regarded as inducible DAMPs because, unlike constitutive DAMPs inherently present in cells, they are actively produced or upregulated by the host cells in response to stress (Tanaka and Heil 2021). Constitutive DAMPs are thought to play a role in maintaining immune surveillance and homeostasis, whereas inducible DAMPs are involved in amplifying immune responses during pathological conditions. However, it should be noted that this categorization is not always followed for animal cytokines, and some phytocytokines can be secreted into the apoplast in the absence of cell damage or before such damage occurs.

Phytocytokines can vary in molecular characteristics, post-translational processing and modifications, secretory routes, and modes of action (Figure 1, Figure 2, Figure 3, Figure 4; Table 1). Upon perception of phytocytokines, specific cell surface-localized leucine-rich repeats receptor-like kinases (LRR-RLKs) often heterodimerize with SOMATIC EMBRYOGENESIS RECEPTOR-LIKE KINASE (SERK) co-receptors, e.g., BRASSINOSTEROID INSENSITIVE 1 (BRI1)-ASSOCIATED RECEPTOR KINASE 1 (BAK1)/SERK3 and SERK4, to either enhance or dampen plant immune responses (Figure 3) (Luo 2012; Hou et al., 2021a; Tanaka and Heil 2021; Rzemieniewski and Stegmann 2022).

### The precursors of systemin and hydroxyproline-rich systemin serve as sources of multiple immune signals

*Solanum lycopersicum* systemin (Sys), a wound-induced peptide signal that mediates systemic resistance against insects, was the first phytocytokine identified in plants, with homologs subsequently identified in several species of the Solanaceae family (Pearce et al., 1991; McGurl et al., 1992; Constabel et al., 1998). Sys is encoded as a precursor (pro-Sys) comprising two pro regions of 178 and 4 amino acids (aa) flanking an 18-aa active Sys peptide (Figure 1; Table 1). Pro-Sys processing at the two aspartate residues flanking the active sequence is performed by SlPHYTASPASE 1 (SlPhyt1) and SlPhyt2, two tomato apoplastic and self-activated subtilisin-like serine proteases (subtilases [SBTs]) (Figure 3; Table 1). SlSBT3 is also proposed as a possible protease involved in pro-Sys processing and defense against the specialist herbivore *Manduca sexta* (Figure 4) (Cedzich et al., 2009; Meyer et al., 2016), producing a biologically active peptide with an extra leucine residue at the N terminus (Leu-Sys). Further trimming by a leucine aminopeptidase (LapA) may be required to obtain fully active Sys (Gu and Walling 2000). Lack of a signal peptide may suggest that pro-Sys is exported into the apoplast through a non-canonical secretion pathway. However, evidence suggesting that the pro-Sys-processing phytaspases are located in the apoplast (Chichkova et al., 2010), coupled with the observation that the precursor exhibits an intracellular localization (nuclear-cytoplasmic) (Narváez-Vásquez and Ryan 2004), implies that the precursor is released and processed in the apoplast following cellular damage in wounded or infected tissues (Figure 2) (Beloshistov et al., 2018). Further studies are needed to clarify how pro-Sys reaches the apoplast.

After secretion and maturation, Sys is perceived by the two LRR-RKs SYSTEMIN RECEPTOR 1 (SYR1) and SYR2 (Wang et al., 2018), triggering a downstream pathway leading to JA biosynthesis and the activation of a set of defense responses such as extracellular alkalization, protease inhibitor production, and ethylene emission (Figure 3) (Pearce et al., 1991; Felix and Boller 1995; Schaller 1998; Sun et al., 2011). Sys is involved in resistance against the noctuid moth *Spodoptera littoralis* and the necrotrophic fungus *Botrytis cinerea* and is also reported to attract natural enemies of insects (Figure 4) (Corrado et al., 2007; Coppola et al., 2015; 2019). A more complex role of pro-Sys has been proposed in plant immunity (Corrado et al., 2016). Intriguingly, the N-terminal pro region can elicit an additional defense pathway involving endogenous PGs and CW-derived OGs (Molisso et al., 2022a; 2022b). Mature Sys, its pro region, and OGs could synergistically collaborate to amplify extracellular immune signals.

A hydroxyproline-rich systemin (HypSys), subsequently identified in *Solanaceae* and *Convolvulaceae*, is similar to Sys in size and function without sharing sequence similarity (Narváez-Vásquez et al., 2007; Chen et al., 2008). Tomato HypSys is encoded as a pre-pro-HypSys precursor of 146 aa containing a signal peptide that directs pro-HypSys into the ER and the canonical secretory pathway (Figures 1 and 2; Table 1). Pro-HypSys is a source of multiple bioactive peptides. In tomato plants, it is processed in the apoplast to release three functional peptide homologs: TomHypSys I (18 aa), TomHypSys II (15 aa), and TomHypSys III (15 aa) (Figure 1; Table 1) (Pearce and Ryan 2003). The processing mechanisms required to release active HypSys peptides are still poorly understood. The three TomHypSys peptides are powerful inducers of defense responses, including extracellular alkalization and protease inhibitor expression, and also enhance resistance to herbivorous insects (Figures 3 and 4) (Pearce and Ryan 2003; Narváez-Vásquez et al., 2007; Pearce 2011).

### SGP-rich pro-peptides: PEPs, PIPs, and SCOOPs

Precursors of plant elicitor peptides (pro-PEPs), PAMP-induced secreted peptides (pro-PIPs), and serine-rich endogenous peptides (pro-SCOOPs) have emerged as key factors in plant immunity (Rzemieniewski and Stegmann 2022). The peptides processed from these precursors belong to the SGP-rich peptide superfamily, characterized by biologically active peptide domains with conserved serine, glycine, and proline residues (Huffaker et al., 2006; Hou et al., 2014; Gully et al., 2019). Whereas pro-PEPs and pro-PIPs are largely conserved in angiosperms, pro-SCOOPs seem to be specific to Brassicaceae (Lori et al., 2015; Gully et al., 2019).

The Arabidopsis genome encodes eight pro-PEPs, and orthologs have been identified in many plant species including maize, rice, potato, and soybean (Huffaker et al., 2006; 2011; Poretsky et al., 2020). *Pro-PEP* genes can be induced by pathogens and elicitors (Huffaker et al., 2006; Logemann et al., 2013; Klauser et al., 2015). Application of PEPs can induce the expression of their precursor genes, thus forming a positive signaling feedback loop (Huffaker et al., 2006). PEP1 (23 aa), the first phytocytokine identified in Arabidopsis, is encoded as an inactive 92-aa pro-PEP1 (Figure 1; Table 1). Lack of a signal peptide suggests that PEP1 is not released into the apoplast through a conventional secretion pathway. In healthy cells, pro-PEP1 is retained on the tonoplast. In damaged and elicited cells, pro-PEP1 maturation can be mediated by Ca^2+^-dependent type II metacaspases (MCs) (MC4–MC9), releasing active PEP1 (Hander et al., 2019; Shen et al., 2019) (Figure 2; Table 1). Once released, mature PEP1 can be perceived on the plasma membrane by the LRR-RKs PEP RECEPTOR 1 (PEPR1)/PEPR2 and the co-receptor BAK1 (Yamaguchi et al., 2006; Schulze et al., 2010). It can be speculated that PEP1 could be released into the cytosol, then move across the compromised plasma membrane by passive diffusion (or active secretion) to bind the extracellular domains of PEPR1/2-BAK1 in surrounding intact cells and activate a defense response (Figures 2 and 3) (Hander et al., 2019). PEP2, a paralog of PEP1, is perceived by PEPR1/2, whereas another paralog, PEP3, is perceived only by PEPR1 (Yamaguchi et al., 2010). How the PEP2 and PEP3 peptides are generated remains to be determined. PEP1 can promote plant resistance to different pathogens such as the bacterium *Pseudomonas syringae*, the fungus *B. cinerea*, and the oomycete *Phytophthora infestans* (Figure 4) (Huffaker et al., 2006; Yamaguchi et al., 2010; Liu et al., 2013; Okada et al., 2020). PEP2 and PEP3 play a role in Arabidopsis resistance to hemibiotrophic bacteria and fungi through the JA/ethylene and SA pathways (Ross et al., 2014; Yamada et al., 2016). CW damage upregulates *pro-PEP1* and *pro-PEP3* expression, whereas the application of PEP1 and PEP3 represses CW-damage-induced JA and SA production (Engelsdorf et al., 2018). These results suggest a cooperation between immune signaling and maintenance of CW integrity in the regulation of defense responses. Evidence that the signaling system mediated by PEPs and their receptors, the PEPRs, contributes to OG-activated immunity (Gravino et al., 2017) highlights the interconnection between different elicitation pathways induced by phytocytokines and CW-derived DAMPs.

Eleven *pro-PIP* genes are present in the Arabidopsis genome (Hou et al., 2014). Pro-PIP1 and pro-PIP2 (72 and 84 aa, respectively) are encoded as precursors containing a signal peptide (pre-pro-PIP) that directs them into the canonical secretory pathway and are processed in the apoplast by unknown protease(s) into active PIP1 (13 aa) and PIP2 (13 aa) (Figure 1; Table 1) (Hou et al., 2014). Their gene expression is induced by several microbial MAMPs such as flg22 (22-aa flagellin peptide), elf18 (18 aa of bacterial elongation factor Tu), and chitin. Overexpression of *pro-PIP1* and *pro-PIP2*, or exogenous application of PIP1 and PIP2 synthetic peptides, enhances immune responses and resistance to *P. syringae* and *Fusarium oxysporum* (Figure 4). PIP1 is perceived by RECEPTOR-LIKE KINASE 7 (RLK7) and shares overlapping but also distinct signaling components with PEP1–PEPR1 (Figure 3). Both PIP1 and PEP2 induce the expression of pro-PEP1, PEPR1, and the flagellin receptor FLAGELLIN-SENSING 2 (FLS2), further amplifying the immune responses mediated by the PAMP flagellin. StPIP1_1, a potato homolog of Arabidopsis PIP1, is secreted to the apoplast, causing an oxidative burst in an StSERK3A/B-dependent manner, and induces defense gene expression as well as defense metabolite accumulation (Nietzschmann et al., 2023). By contrast, PIP3 was proposed to attenuate immunity (Najafi et al., 2020). Pro-PIP3-overexpressing plants exhibit higher susceptibility to both the necrotrophic pathogen *B*. *cinerea* and the hemibiotrophic pathogen *P*. *syringae*. Simultaneous activation of the SA and JA pathways in PIP3-overexpressing plants could prioritize SA, facilitating cell death and necrotrophic colonization.

Recently, at least 50 SCOOP isoforms have been identified in the Arabidopsis genome (Yang et al., 2023). The best-characterized member is encoded by *SCOOP12* as a pre-pro-SCOOP12 precursor (78 aa) containing a signal peptide, a pro region (19 aa), and the mature peptide (35 aa) (Hou et al., 2021b) (Figure 1; Table 1). SCOOP12 shares, with several other members, a 13- to 15-aa conserved epitope that includes an “SxS” motif that is essential for receptor recognition and bioactivity (Gully et al., 2019; Hou et al., 2021b; Rhodes et al., 2021). A 13-aa peptide is defined as the minimal active epitope for SCOOP12 (Rhodes et al., 2021; Yang et al., 2023). The extracellular protein SBT3.5 was recently identified as the protease that processes pro-SCOOP12 (Table 1) (Yang et al., 2023). Interestingly, SBT3.5 can also process the precursor of PME17, regulating PME activity and pectin methylesterification in plant immunity against *B. cinerea* (pro-PMEs are discussed later) (Senechal et al., 2014; Del Corpo et al., 2020; Coculo et al., 2023). This implies a shared activation of phytocytokines and enzymes participating in CW remodeling and signaling in plant immunity. After processing, SCOOP12 is sensed by MALE DISCOVERER 1-INTERACTING RECEPTOR-LIKE KINASE 2 (MIK2), triggering formation of the MIK2–BAK1 complex (Figure 3) (Hou et al., 2021b; Rhodes et al., 2021) and activating immune responses that lead to resistance to *P. syringae*, *F. oxysporum*, and *S. littoralis* but susceptibility to *Erwinia amylovora* (Figure 4) (Gully et al., 2019; Hou et al., 2021b; Rhodes et al., 2021; Stahl et al., 2022). SCOOP24–28 are also able to activate immune responses in Arabidopsis (Zhang et al., 2022a), with SCOOP27 also triggering Arabidopsis resistance to the vascular wilt fungus *F. oxysporum*.

### RALFs in plant immunity: Pros and cons

Another important family of phytocytokines is represented by the rapid alkalinization factors (RALFs) (Zhang et al., 2023), which are cysteine-rich peptides that induce apoplastic alkalization and serve as key regulators of plant growth, fertility, and immunity (Ge et al., 2017; Mecchia et al., 2017; Blackburn et al., 2020; Moussu et al., 2023). Phylogenetic analysis has revealed that the RALF family has diverged into four clades (Campbell and Turner 2017). Clades I, II, and III exhibit conserved structural features crucial for their function, including four conserved cysteine residues involved in disulfide bond formation for proper peptide folding (Frederick et al., 2019), YIXY motifs essential for biological activity (Pearce et al., 2010), and a di-basic motif (RRXL) recognized and cleaved by SBT6.1 (also known as site-1 protease, S1P) to generate the mature peptide (Matos et al., 2008). RALF precursors (pre-pro-RALFs) belong to these clades and typically consist of 80- to 120-aa proteins (He et al., 2022a). By contrast, clade IV proteins, previously referred to as RALF-related proteins, lack all characteristic RALF features, with most members lacking the RRXL motif (Campbell and Turner 2017). The Arabidopsis RALF family comprises more than 30 members (Abarca et al., 2021). For example, pre-pro-RALF23 is a 138-aa precursor processed by SBT6.1 to release the 60-aa pro region from the 50-aa active domain (Figure 1; Table 1) (Srivastava et al., 2009). Because RALFs are predicted to be secreted peptides and SBT6.1 is localized in the Golgi, pro-RALF23 is likely processed in this compartment during its secretion pathway (Figure 2) (Liu et al., 2007; Srivastava et al., 2009). It has also been speculated that some SBT6.1 may be secreted and that pro-RALFs could be proteolytically processed in the apoplast (Srivastava et al., 2009). Accordingly, a RALF precursor was found in the ER and later in the apoplast in *Nicotiana benthamiana* leaves (Escobar et al., 2003).

RALFs are ligands of FERONIA (FER), a receptor that belongs to the 17-member CATHARANTHUS ROSEUS RECEPTOR-LIKE KINASE 1-LIKE (CrRLK1L) family and regulates multifaceted functions in growth, development, and responses to environmental factors and pathogens (Figure 3) (Haruta et al., 2014; Franck et al., 2018; Zhang et al., 2020; Malivert and Hamant 2023; Cheung 2024). For instance, FER, together with the co-receptors LORELEI (LRE)-LIKE GLYCOSYLPHOSPHATIDYLINOSITOL (GPI)-ANCHORED PROTEIN 1 (LLG1), is required for flg22-induced FLS2–BAK1 complex formation (Stegmann et al., 2017; Xiao et al., 2019). Conversely, RALFs that harbor AtS1P-cleavage sites, such as RALF23 and RALF33, negatively regulate immunity to hemibiotrophic pathogens (Stegmann et al., 2017; Merino et al., 2019). Indeed, in the presence of RALF23, a heterotrimeric RALF23–FER–LLG1 heterocomplex is formed (Stegmann et al., 2017; Xiao et al., 2019), leading to an alteration of the plasma membrane nanoscale organization of FLS2 and BAK1, inhibiting both flg22-induced FLS2–BAK1 complex formation and flg22-induced immunity (Gronnier et al., 2022). Recently, it was discovered that binding of RALF1 and RALF23 to de-methylesterified OGs leads to RALF–pectin phase separation, pectin–RALF–FER–LLG1 condensation, and induced global endocytosis of clusters of receptors involved in immunity and development, which may explain the inhibition of PTI (Liu et al., 2024). These observations provide functional evidence for the previously proposed link between RALFs, FER, pectins, OGs, and CW integrity (Feng et al., 2019; Moussu et al., 2023; Rößling et al., 2023; Schoenaers et al., 2023; Zhou et al., 2024). Furthermore, RALF-induced endocytosis is abolished in the presence of OGOX1 (Liu et al., 2024), an OG oxidase that can inactivate OG signaling (Benedetti et al., 2018; De Lorenzo and Cervone 2022). The function of the pectin–RALF complex in plant–microbe interactions and the role of pro-RALF processing have only begun to be unraveled. RALF17, which lacks the processing site and the pro region, unlike RALF1 and RALF23, induces immune responses in an FER-dependent manner. The presence of a pro region and a processing site does not seem to be the sole determinant for predicting the functions of RALFs in plant immunity (He et al., 2023). Indeed, RALF22, an RRXL-type RALF with an S1P site, acts similarly to the non-RRXL-type RALF17, eliciting various immune responses and resistance against the necrotrophic fungal pathogen *Sclerotinia sclerotiorum* in an FER-dependent manner in Arabidopsis and other *Brassica* crops, including *Brassica napus*, *B. pekinensis*, and *B. campestris* (Figure 4) (He et al., 2023). RALF22 also amplifies the PEP3-induced immune signal by dramatically upregulating *pro-PEP3* expression, suggesting an amplification effect mediated by different phytocytokines (He et al., 2023).

### Sulfated plant peptide hormones: GOLVENs, phytosulfokines, and PSYs

Several classes of peptides containing tyrosine-sulfated aa are signals in plant immunity and development, perceived by LRR-RLKs of classes X and XI (Kaufmann and Sauter 2019). The root meristem growth factors (RGFs), also known as CLE-like (CLEL) or GOLVEN (GLV) peptides (RGF/CLEL/GLV), share structural similarities with CLE peptides (discussed later) and were initially identified as essential factors for root meristem maintenance and gravitropism (Matsuzaki et al., 2010; Whitford et al., 2012). Some of the 11 RGFs/GLVs in Arabidopsis are encoded as pre-pro-proteins and subsequently processed via post-translational sulfation and proteolysis to release C-terminal 12- to 15-aa biologically active peptides. This is the case for pre-pro-RGF7/GLV4 and pre-pro-RGF9/GLV2 (Figure 1; Table 1). Mature RGF7/GLV4 is perceived in the apoplast by the RGF1 INSENSITIVE (RGI)-family receptors RGI4 and RGI5 (Wang et al., 2021d), whereas RGF9/GLV2 is perceived by RGI3 (Stegmann et al., 2022) (Figure 3). Both peptides contribute to the induction of defense responses and resistance to *P. syringae* (Figure 4). Phytosulfokines (PSKs) are short (5-aa) tyrosine-sulfated peptides involved in numerous processes of plant growth, development, and immunity (Yang et al., 2001; Ladwig et al., 2015; Zhang et al., 2018). Arabidopsis PSK1 and PSK4 are encoded as pre-pro-precursors of 87 and 79 aa, respectively (Figure 1; Table 1). Pro-PSK1 is processed by SBT3.8 (Stührwohldt et al., 2021), whereas pro-PSK4 is cleaved by SBT1.1 (Figure 3) (Srivastava et al., 2008). The enzyme tyrosylprotein sulfotransferase (TPST) sulfates the tyrosine residues on pro-PSKs (Hanai et al., 2000; Komori et al., 2009). Whereas pro-PSKs and related SBTs are extracellular proteins, TPST is Golgi localized (Srivastava et al., 2008; Komori et al., 2009; Stührwohldt et al., 2020; 2021). PSK likely undergoes sulfation by TPST in the Golgi before being secreted into the apoplast for cleavage by a specific SBT to produce a mature PSK peptide. Both active peptides are perceived by PSK RECEPTOR 1 (PSKR1), leading to attenuation of immune responses and SA signaling and increased susceptibility to biotrophic and hemibiotrophic pathogens such as *Hyaloperonospora arabidopsidis* and *P. syringae* (Figures 3 and 4) (Igarashi et al., 2012; Rodiuc et al., 2016). PSKR1 interacts with the Ca^2+^-dependent protein kinase CPK28, which phosphorylates glutamine synthetase GS2 at two sites (serine-334 and serine-360), regulating plant defense and growth, respectively (Ding et al., 2023). On the other hand, PSK1 and PSK4 also activate JA signaling, enhancing resistance to necrotrophs such as *Alternaria brassicicola* (Amano et al., 2007; Mosher et al., 2013; Zhang et al., 2018). In tomato, pro-PSKs can be processed by SlPhyt2 (Reichardt et al., 2020). SlPSKR1-mediated PSK signaling increases cytosolic Ca^2+^ concentration and enhances tomato immunity to *B. cinerea* (Zhang et al., 2018). SlPSKR1 is regulated by U-box E3 ligases PUB12/13 via ubiquitylation (Hu et al., 2023). PSKs inhibit protein degradation of PSKR1 by PUB12/13, contributing to immunity to *B. cinerea*. In cotton, OG treatment significantly increases the expression of PSK, which can in turn induce PME inhibitor 13 (*GbPMEI13*) and increase resistance to *Verticillium dahliae* (Zhang et al., 2022b). *GbPMEI13* can inhibit *V. dahliae* mycelial growth and, by increasing pectin methylesterification, protect pectin degradation from *V*. *dahliae* PGs. An antagonistic effect emerges for PSK-mediated signaling, favoring biotrophs and counteracting necrotrophs. In resistance to necrotrophs, CW DAMPs can amplify PSK signaling, leading to pectin remodeling and CW reinforcement.

Arabidopsis PLANT PEPTIDE CONTAINING SULFATED TYROSINE 1 (PSY1), a functional analog of PSKs, is a sulfated and triply arabinosylated 18-aa peptide synthesized as an inactive 75-aa pre-pro-PSY1 (Figure 1; Table 1). After processing mediated by still-unknown proteases, an active 18-aa peptide is released, which suppresses immune responses and compromises resistance to *P. syringae* and *F. oxysporum* but improves response to *A. brassicicola* (Figure 4) (Mosher et al., 2013; Shen and Diener 2013). A recent study revealed that three PSY receptors (PSYRs), PSYR1, PSYR2, and PSYR3, act as direct ligand receptors for the PSY family peptides (PSY1–PSY9), potentially mediating the trade-off between plant growth and stress response (Ogawa-Ohnishi et al., 2022). PSKs and PSY1 may be particularly useful for genetic programs aimed at improving protection against necrotrophic pathogens but could compromise the response to hemibiotrophs. Interestingly, PSY1 can activate expression of Arabidopsis genes involved in CW modification, including PMEs (discussed later) (Mahmood et al., 2014).

### ZYP1 contributes to protection of maize against biotrophs

*ZEA MAYS* IMMUNE SIGNALING PEPTIDE 1 (ZIP1) was identified as a phytocytokine that protects maize against biotrophs (Ziemann et al., 2018). pro-ZIP1 (137 aa) is processed by the apoplastic maize papain-like cysteine proteases (PLCPS) CP1 and CP2 (described later) to release the 17-aa active peptide (Figure 1; Table 1). Evidence that pro-ZIP1 lacks a signal peptide and that CPs are apoplastic proteases suggests that the precursor is not secreted through the conventional protein secretion pathway (Figure 2). ZIP1 strongly activates SA-dependent signaling, promoting efficient defense activation against biotrophic pathogens but potentially facilitating colonization by necrotrophic pathogens that may benefit from cell death (Spoel et al., 2007). Consistent with these observations, ZYP1 was found to promote maize resistance to the biotrophic fungus *Ustilago maydis* but enhance susceptibility to the necrotrophic fungus *B. cinerea* (Figure 4) (Ziemann et al., 2018). Pro-ZYP1 has recently been identified as an orphan gene that evolved *de novo* from a retrotransposon (Depotter et al., 2022).

### IDA as a defense guard for cell separation processes

INFLORESCENCE DEFICIENT IN ABSCISSION (IDA) and IDA-Like (IDL) are small peptides encoded as precursors that carry an N-terminal signal peptide, a pro region, and a C-terminal extended PIP domain (Figure 1; Table 1) (Stenvik et al., 2008). SBT4.12, SBT4.13, and SBT5.2 cleave the pro regions, releasing the IDA active peptide (Schardon et al., 2016). IDA can be perceived by HAESA (HAE) and HAESA-LIKE 2 (HSL2) receptors (Figure 3) (Santiago et al., 2016). IDL6 and IDL7 are negative modulators of stress-induced ROS signaling in Arabidopsis (Vie et al., 2017). Intriguingly, IDL6 promotes the expression of ADPG2, a plant PG that increases pectin degradation and enhances *P. syringae* infection (Figure 4) (Wang et al., 2017b). Although this plant PG has been linked to susceptibility, its production could also represent an attempt by the plant to produce OGs active as DAMPs that, however, are not sufficient to reverse the outcome of the infection. A recent study proposed that the IDA–HAE/HSL2 signaling pathway can positively modulate defense responses as a precaution against pathogen attack in tissues subjected to cell separation, a process largely influenced by pectin structure (Lionetti et al., 2010; 2014a; Lalun et al., 2023). All these findings highlight a possible relationship between IDL6-mediated responses and pectin degradation in plant immunity that deserves further investigation.

### The cryptic CAPE peptide in PR1 proteins

Plant pathogenesis-related (PR) proteins fall into 17 distinct classes (PR1–PR17) and are strongly induced by biotic and abiotic stresses (van Loon et al., 2006). Proteins of the PR1 class have been identified in many different plant species and are among the founding members of the CAP (cysteine-rich secretory protein [CRISP], antigen 5 [Ag5], and pathogenesis-related 1 protein) superfamily (Fraser 1981; Gibbs et al., 2008; Shin et al., 2014). Hidden within the PR1 precursors, stress-related peptides named CAP-derived peptides (CAPEs) have been discovered (Chen et al., 2014; Chien et al., 2015; Breen et al., 2016). Arabidopsis PR1 is encoded as a 161-aa pre-pro-protein containing a predicted 124-aa pro region and the 11-aa active CAPE9 (Figure 1; Table 1) (Chien et al., 2015). Maturation of the pro-protein is mediated by xylem cysteine peptidase 1 (XCP1), a papain-like protease (described later) that recognizes and cleaves the conserved CNYx motif, releasing CAPE9 (Chen et al., 2023b). Because PR1 and XCP1 co-localize in the apoplast, it is possible that CAPE9 production takes place in the extracellular space (Chen et al., 2023b). CAPE9 regulates SA levels and enhances resistance to *P. syringae* in Arabidopsis (Figures 3 and 4) (Chen et al., 2023b). Processing of tomato PR-1b into SlCAPE1 (SlCAPE1) was associated with apoplastic aspartic proteases (APs) (Rodrigo et al., 1991; Chen et al., 2014). SlCAPE1 production can be induced by wounding and activates expression of multiple defense-related genes, induction of SA and JA biosynthesis, and enhancement of resistance to herbivorous *Spodoptera litura* larvae and *P. syringae* (Chen et al., 2014).

### Some phytocytokine receptors function in immunity, but what about the role of their cognate peptides?

Here, we mention two classes of peptides not yet directly involved in immunity but sensed by receptors clearly associated with plant stress, thus making them interesting for future investigations. CLAVATA3/EMBRYO-SURROUNDING REGION (CLEs) play important roles in various developmental and physiological processes and are considered peptide hormones (Willoughby and Nimchuk 2021). Currently, 32 CLE genes have been identified in Arabidopsis, and various numbers of CLEs are found in all species. CLAVATA 3 (CLV3) was the first CLE identified and is involved in meristem maintenance (Figure 1; Table 1) (Hirakawa 2021). CLV3 is encoded as a pre-pro-CLV3 precursor (96 aa) and contains two pro regions of 48 and 14 aa flanking the mature active CLE domain of 12–13 hydroxylated and glycosylated aa. Pro-CLV3 undergoes very rapid intracellular processing in the early compartments of the secretory pathway before being secreted into the apoplast (Rojo et al., 2002; De Marchis et al., 2018). The LRR receptor kinase CLV1 and the complex formed by the LRR receptor-like protein CLV2 and the transmembrane pseudo-kinase CORYNE are receptors of CLV3 (Figure 3) (Müller et al., 2008). Both CLV1 and CLV2 can act as susceptibility factors for *Ralstonia solanacearum*, *H. arabidopsidis*, and *Heterodera schachtii* (Replogle et al., 2011; Hanemian et al., 2016). On the other hand, *clv1* mutants are more susceptible than WT plants to *P. syringae*, *B. cinerea*, and *Plectrosphaerella cucumerina*, indicating that CLV1 can contribute to plant defense in response to specific pathogens (Hanemian et al., 2016). Whether the CLV3 peptide itself can influence immunity is not yet known.

EPIDERMAL PATTERNING FACTOR (EPF) and EPF-LIKE (EPFL) are a family of small secreted pre-pro-peptides involved in many aspects of plant growth and development (Hara et al., 2007; Rychel et al., 2010). EPF1 is synthesized as an inactive 122-aa pre-pro-peptide with a signal peptide and a 22-aa pro region. After maturation, the 74-aa active peptide is released into the apoplast and controls stomatal density and patterning by regulating asymmetric cell division (Hara et al., 2007). EPF2 affects leaf stomatal density during leaf development (Xiong et al., 2022). Although EPF/EPFLs have never been directly implicated in plant immunity, their receptor ERECTA promotes plant resistance to pathogens with different lifestyles, including the necrotrophic or hemibiotrophic fungi *P. cucumerina*, *Verticillium longisporum*, and *Magnaporthe oryzae*, the bacterium *R. solanacearum*, the oomycete *Pythium irregulare*, etc. (Godiard et al., 2003; Llorente et al., 2005; Häffner et al., 2014). Intriguingly, ERECTA was hypothesized to act as a CW integrity sensor of CW DAMPs released during infection (Sánchez-Rodríguez et al., 2009).

## Maturation of CW remodeling enzymes contributes to CW maintenance and immunity

Plants have developed monitoring systems to transmit the state of perturbed CW structure (Rui and Dinneny 2020; Baez et al., 2022). Receptor-like kinases such as HERKULES1 (HERK1), THESEUS (THE1), FER, WALL-ASSOCIATED KINASE1 (WAK1), KINESIN 13A (KIN-13A), FEI1, FEI2, and RESISTANCE TO FUSARIUM OXYSPORUM 1 (RFO1) act as molecular sentinels of CW integrity, some binding directly to CW components with their highly specialized ectodomains (Figure 3) (Decreux and Messiaen 2005; Xu et al., 2008; Guo et al., 2009; Oda and Fukuda 2013; Merz et al., 2017; Feng et al., 2018; Huerta et al., 2023). Although many of these receptors sense wall integrity during development, emerging evidence implicates some of these sensors in abiotic and biotic stress responses (Brutus et al., 2010; Qu et al., 2017; Guo et al., 2018; Gigli-Bisceglia et al., 2022; Huerta et al., 2023).

Plant cells attempt to maintain CW integrity during plant–microbe interactions (Seifert and Blaukopf 2010; Rui and Dinneny 2020). CW reinforcement and remodeling can occur through changes in pectin esterification, crosslink formation between pectin polysaccharides and other glycans and/or phenols, hemicellulose feruloylation, callose deposition, and secretion of structural proteins such as extensins and arabinogalactan proteins (Pena et al., 2004; Rashid 2016; Reem et al., 2016; Lionetti et al., 2017). CW remodeling enzymes represent a significant portion of the plant CW proteome (Albenne et al., 2014). These proteins typically possess a signal peptide for translocation into the ER, and some are synthesized as inactive zymogens (Figure 1; Table 1). Upon activation, these enzymes mediate fine structural remodeling of CW polysaccharides to strengthen the physical barrier against pathogens and may trigger specific immune signaling pathways (Bellincampi et al., 2014; Swaminathan et al., 2022; Coculo et al., 2023). Moreover, several lines of evidence link the processing of precursors of CW-remodeling enzymes and the release of their modifying activity with the activation, perception, and signaling of the phytocytokines discussed previously.

### Pectin remodeling and the generation of danger signals: The key roles of pro-PMEs

HG is a linear pectin polymer of galacturonic acid (GalA) monomers synthesized in the Golgi complex and delivered to the CW in a highly methylesterified form (Goubet and Mohnen 1999; Ibar and Orellana 2007). The degree of HG methylesterification is an important biochemical trait in plant defense against pathogens (Lionetti et al., 2007; 2012; Raiola et al., 2011; Coculo et al., 2023) and is controlled *in muro* by PMEs (Figure 3) (Pelloux et al., 2007). PMEs catalyze the hydrolysis of methyl ester bonds at the C-6 of Gal A residues in the apoplast, producing acidic pectins with negatively charged carboxyl groups and releasing methanol (MeOH) and protons. Arabidopsis PMEs are classified into two groups based on their structures. Group 1 is composed of 21 isoforms that in the mature form comprise only the active catalytic domain. Group 2 consists of 45 isoforms synthesized as pro-PMEs, zymogens organized as a polycistronic messenger RNA resembling an operon-like gene cluster (Coculo and Lionetti 2022). In pro-PMEs (also referred to as PMEI-PMEs), the pro region shares structural similarities with PMEIs and can act as an intramolecular inhibitor (Del Corpo et al., 2020).

A local and strong induction of plant PME activity is triggered in Arabidopsis upon infection with a wide range of pathogens, including fungi such as *B. cinerea* and *A. brassicicola*, bacteria such as *P. syringae*, and viruses such as the *Turnip vein-clearing virus* (TVCV) (Bethke et al., 2014; Lionetti et al., 2014b; 2017). Intriguingly, all PME isoforms expressed in Arabidopsis infected with *B. cinerea* are pro-PMEs (Coculo et al., 2023). Specifically, *AtPME17* is induced during infection with a wide range of pathogens and can be considered a general biomarker for pathogenesis (Lionetti et al., 2012). pro-PME17 is a well-characterized zymogen of 511 aa composed of a 148-aa pro region, a 33-aa linker sequence with a protease cleavage site, and a 307-aa active domain (Figure 1; Table 1) (Del Corpo et al., 2020). The enzyme is secreted into the apoplast through the conventional ER–Golgi secretion pathway (Coculo et al., 2023) (Figure 2). PME17 triggers defense-related PME activity and resistance to *B. cinerea* and *P. syringae* (Bethke et al., 2014; Del Corpo et al., 2020). There is evidence linking the activity of some SBTs to the maturation of specific pro-PMEs in plant growth and development (Rautengarten et al., 2008; Wolf et al., 2009; Senechal et al., 2014). Specifically, SBT3.3 and SBT3.5 have been identified as promoters of defense-related PME activity (Figure 3) (Coculo et al., 2023). Interestingly, SBT3.5 has also been found to cleave pro-SCOOP12 to release active SCOOP12 (Yang et al., 2023), suggesting a potential role for SBT3.5 as a common switch for both PME and phytocytokines to enhance defense responses against pathogens.

The activity of plant PMEs, in cooperation with PGs and polygalacturonase-inhibiting proteins, can influence immunity by favoring the release of OGs with different degrees of methylesterification (Osorio et al., 2008; 2011; Voxeur et al., 2019; Xiao et al., 2024). De-methylesterification of pectin by PMEs also represents the primary mechanism for generation of plant-derived MeOH, a DAMP-like warning signal (Dorokhov et al., 2012; Hann et al., 2014; Komarova et al., 2014). OGs and MeOH can be considered PME-related DAMPs. The processing of pro-PME may represent a strategy for prompt triggering of OG and MeOH production in the apoplast. Moreover, PMEs, by performing block-wise pectin de-methylesterification, can produce negatively charged HG regions, inducing Ca^2+^-mediated crosslinks and thereby strengthening the CW and hindering pathogen penetration (Del Corpo et al., 2020). Blockwise de-methylesterification of pectin by PMEs may also facilitate the binding of cell surface sensors to pectin, favoring immune signaling pathways. Receptors such as WAK1, WAK2, and FER preferentially bind to de-methylesterified pectins (Lin et al., 2022), with WAK1 acting as a sensor of OGs for the activation of immune responses in Arabidopsis (Figure 3) (Brutus et al., 2010). Furthermore, as described previously, the interaction between RALF peptides and de-methylesterified OGs can initiate RALF-triggered cell surface responses (Liu et al., 2024). An interesting possibility is that concomitant maturation of PME and RALF precursors may activate this physiological process.

Defense-related PME activity also requires post-transcriptional regulation by PMEI (Coculo and Lionetti 2022). PMEI10, PMEI11, and PMEI12 are mediators of CW integrity maintenance in Arabidopsis immunity to *B. cinerea* at advanced infection stages (Lionetti et al., 2017; Coculo and Lionetti 2022). This is because high methylesterification can shield pectin from degradation by PGs, helping plants to resist pathogenic fungal infections (Lionetti et al., 2007; 2017; Liu et al., 2018b). *PMEI11* expression is controlled by OGs and other elicitors, suggesting a possible OG-regulated feedback loop in the post-transcriptional regulation of PME activity.

### Activation of xylosidases and arabinofuranosidases triggers plant defense against pathogens

Hemicelluloses and other pectic components, apart from HG, can also be modified by CW remodeling enzymes during development and microbial attack, resulting in the release of oligosaccharides (Fry et al., 1993). Xylan, a heteropolysaccharide with a glycosidic structure of β-(1,4)-linked D-xylose, is often branched with side chains consisting of arabinose, glucuronic acid, and other groups (Scheller and Ulvskov 2010). Degradation of arabinoxylan requires the coordinated action of several degradative enzymatic activities (Minic et al., 2004). β-D-Xylosidases (XYLs) hydrolyze xylose oligosaccharides previously produced during xylan degradation by xylanases (Qing et al., 2010; Qing and Wyman 2011). Debranching enzymes such as the bifunctional α-L-arabinofuranosidase/β-D-xylosidase (ARA-I), along with other hemicellulolytic enzymes, remove arabinose side chains to control degradation (Figure 3) (Matsuo et al., 2000). Barley XYL and ARA-I are both predicted to be synthesized as 777-aa zymogens (pre-pro-XYL and pre-pro-ARA-I) with signal peptides (Lee et al., 2003). Both precursors undergo proteolytic maturation by removal of 130 aa from the C terminus (Figure 1; Table 1). These enzymatic activities could contribute to the release of arabinoxylan oligosaccharides, known as functional CW DAMPs (Mélida et al., 2020). Apoplastic β-D-XYLOSIDASE 4 (BXL4), also referred to as XYL4, a homolog of ARA-I in Arabidopsis, likely possesses xylosidase and arabinosidase activities (Minic et al., 2004; Arsovski et al., 2009) and contributes to immunity against *B. cinerea* and *P. syringae* (Figure 4) (Guzha et al., 2022; Bauer et al., 2023). RG-I is a pectic heteropolysaccharide consisting of the repeating disaccharide unit (1,2) α-L-rhamnosyl-(1,4) α-GalA, characterized by arabinan, galactan, arabinogalactan, and xylan side chains (Mohnen 2008; Ralet et al., 2016; Amos et al., 2022). BXL4 could trim the side chains of RG-I, likely creating more space for increased crosslinking of pectin by Ca^2+^ (Moore et al., 2008), thus improving CW recalcitrance to fungal penetration. In addition, CW remodeling and/or the oligorhamnogalacturonides, RG-I fragments potentially released by BXL4, could be perceived by cell surface sensors, resulting in activation of plant defense responses (Claverie et al., 2018). BXL4 is involved in systemic acquired resistance, a long-distance signaling mechanism that provides broad-spectrum and long-lasting protection against secondary infections (Durrant and Dong 2004; Breitenbach et al., 2014; Guzha et al., 2022).

## Pro-proteases involved in generation of extracellular danger signals in plant immunity

Proteases, along with their substrates and inhibitors, are categorized in the MEROPS database (https://www.ebi.ac.uk/merops/) (Rawlings et al., 2018). On the basis of the hydrolyzing aa in the active site and the mechanism of peptide bond cleavage, proteases can be classified into seven main classes: aspartic, cysteine, glutamic, metallic, serine, asparagine, and threonine proteases. Proteases play crucial roles in plant immunity, including the processing of pro-peptides and zymogens (Figueiredo et al., 2018; Schaller et al., 2018; Godson and van der Hoorn 2021). Interestingly, proteases themselves can be synthesized as pro-enzymes that require maturation to release the active protein. The processing of protease precursors can be autocatalytic and require specific compartment conditions and additional factors, and it can also be performed by other proteases as part of a proteolytic cascade (Paulus and Van der Hoorn, 2019; Paulus et al., 2020). In this review, we focus on protease precursors that, once processed, mediate the release of danger signals in the apoplast.

### Subtilisin-like serine proteases

SBTs constitute the second largest family of serine peptidases and are widespread among all living organisms (Rose et al., 2010). By targeting proteins and peptides, plant SBTs participate in a broad spectrum of biological functions, including stomatal and seed development (Berger and Altmann 2000; Rautengarten et al., 2008), shoot apical meristem maintenance (Liu et al., 2009), and responses to biotic and abiotic stresses (Tornero et al., 1997; Liu et al., 2007). The six genes of the tomato cluster SBT P69 (P69A–P69F) encode proteins potentially involved in plant defense (Paulus et al., 2020). P69B and other SBTs can process pro-Rcr3, a secreted PLCP (discussed later). SBTs trigger an activation cascade to generate and enhance the effectiveness of warning signals, as demonstrated for caspases during programmed cell death in mammals (Paulus and Van der Hoorn 2019). The processing of an extracellular matrix–associated LRR protein by P69C (Tornero et al., 1996) was speculated to mediate molecular recognition to initiate immune signaling.

Several SBTs are synthesized as protein precursors that are activated through an autocatalytic process at both N and C termini (Cedzich et al., 2009; Plattner et al., 2014). In Arabidopsis, the SBT family comprises 56 members distributed in six distinct subgroups (SBT1–6) (Rautengarten et al., 2005). SBT3.3 is an ortholog of tomato P69C in Arabidopsis. It is synthesized as a pre-pro-SBT3.3 (777 aa) with a signal peptide preceding the 87-aa pro region and 666-aa secreted active protease (Figure 1; Table 1). SBT3.3 is secreted into the apoplast through an unconventional secretion pathway involving a double-membrane exocyst-positive organelle (Figure 2) (Coculo et al., 2023). As discussed previously, its substrate pro-PME17 follows the conventional secretion pathway. It is possible that the spatial separation during secretion of SBTs and PMEs is necessary to prevent premature and intracellular activation of PMEs, which could lead to pectin crosslinking and gelation in the Golgi. SBT3.3 overexpression confers enhanced MAPK activation and enhanced disease resistance to *P. syringae* and *H. arabidopsidis* (Ramírez et al., 2013). SBT3.3 also induces “priming,” a sensory state that makes the plant capable of inducing faster and stronger defense responses. SBT3.3 promotes PME activity and pectin de-methylesterification for Arabidopsis immunity against *B. cinerea* (Figure 4) (Coculo et al., 2023). Moreover, SBT3.3 expression responds to H_2_O_2_ and is induced by OGs and MAMPs (Ramírez et al., 2013; Coculo et al., 2023), and SBT3.3 overexpression over-activates specific defense-related genes, including *WRKY33* and *PAD3* involved in camalexin accumulation and *WAK2* involved in maintenance of pectin integrity (Birkenbihl et al., 2012; Kohorn et al., 2014). It is conceivable that SBTs can activate PME activity and pectin integrity signaling for a timely immune response.

### PLCPs

PLCPs constitute a large multigenic family (31 isoforms in Arabidopsis) involved in several biological processes, including plant growth, seed germination, anther development, senescence, immunity, and stress responses (Misas-Villamil et al., 2016; Liu et al., 2018a). These proteases are synthesized as zymogens with an autoinhibitory pro region (pre-pro-PLCPs). For example, Arabidopsis responsive-to-desiccation-21 (RD21) is synthesized as a 462-aa pre-pro-RD21 consisting of an N-terminal pro region of 115 aa, the 216-aa active enzyme, and a C-terminal region of 110 aa organized into a proline-rich domain and a granulin domain (Figure 1; Table 1) (Shindo et al., 2012). Pro-RD21 undergoes extensive post-translational processing, the details of which require further investigation (Gu et al., 2012). RD21 was detected in the vacuole and ER bodies and can be fucosylated in the Golgi, trafficked in the lytic vacuoles, and later released into the apoplast (Hayashi et al., 2001; Yamada et al., 2001; Andème Ondzighi et al., 2008; Gu et al., 2012). It is conceivable that multivesicular bodies (also known as pre-vacuolar compartments or late endosomes) mediate the secretion of RD21 into the apoplast following pathogen attack (Figure 2) (Cui et al., 2016). Pro-RD21 contributes to immunity against *B. cinerea* (Figure 4) (Shindo et al., 2012). Silencing the *RD21* ortholog in *N. benthamiana* increases susceptibility to *P. infestans* (Kaschani et al., 2010). Interestingly, RD21 interacts with proteins related to CW modification, including expansin B3 (EXPB3), BXL6, SBT1.7, and glycosyl hydrolase family 38 (α-mannosidase) (GlyH), suggesting a potential role of this protease in modulating CW properties (Pogorelko et al., 2019).

As discussed previously, the maize PLCPs CP1 and CP2 can process pro-ZIP1 to release immune signaling peptides in the apoplast (Ziemann et al., 2018), and XCP1 can cleave PR1 into CAPE9 for systemic immunity in Arabidopsis (Chen et al., 2023b). The tomato PLCP Pro-Rcr3 can be processed by the SBT P69B (also known as pathogenesis-related 7 [PR7]) and other SBTs (Paulus et al., 2020). The active form of Rcr3 contributes to defense against the fungal pathogen *Cladosporium fulvum* and the oomycete late-blight pathogen *P. infestans* (Dixon et al., 2000; Song et al., 2009).

### APs

APs are characterized by the Asp-Gly-Thr aa triad at the active site (Figueiredo et al., 2021). Most Arabidopsis APs are predicted to be extracellular proteases (Wang et al., 2019). Plant APs are synthesized as pre-pro-proteases and participate in a wide range of biological activities, including growth, development, and stress responses (Figueiredo et al., 2021). Arabidopsis constitutive disease resistance 1 (pre-pro-CDR1) is an extracellular AP involved in plant–microbe interactions (Xia et al., 2004; Simões et al., 2007). This protease is synthesized as a 437-aa pre-pro-enzyme comprising an N-terminal pro region of 48 aa and an active enzyme of 364 aa (Figure 1; Table 1). The mechanisms of CDR1 processing remain to be characterized. Arabidopsis *CDR1-D* plants (an activation tagging line) exhibit increased accumulation of PR proteins and enhanced resistance against *P. syringae* (Simões et al., 2007). Overexpression of rice *OsCDR1* leads to constitutive activation of defense responses in both rice and Arabidopsis (Prasad et al., 2009), resulting in increased resistance of Arabidopsis to *P. syringae* and *H. arabidopsidis* and of rice to *Xanthomonas oryzae* and *M. oryzae*. However, the natural substrates for CDR1 are still unknown. Apoplastic APs in both tobacco and tomato have been associated with degradation of PR proteins (Rodrigo et al., 1991). Indeed, APs can cleave tomato PR-1b, a process that may favor the release of the active SlCAPE1 (Rodrigo et al., 1991; Chen et al., 2014).

## Plant proteases convert PAMP or effector precursors into immunogenic elicitors

PAMPs can be concealed within precursors. Hosts can perform precursor modifications and fragmentations for elicitor detection by PRRs (Buscaill and van der Hoorn 2021; Chen et al., 2023a). For instance, plants can deglycosylate flagellin using apoplastic β-galactosidase 1 and subsequently fragment it with an unknown protease to expose the immunogenic flg22 peptide, enhancing its recognition by FLS2 (Buscaill et al., 2019). Successful pathogens may secrete effectors to counteract PTI (Jones and Dangl 2006; Pogorelko et al., 2019). However, some effectors can be recognized by host resistance (R) proteins, leading to effector-triggered immunity (Tsuda et al., 2013; Wang et al., 2021b; Remick et al., 2023). Pathogens also produce effector precursors that require post-translational processing by microbial proteases to generate the active molecules (Van Kan et al., 1991; Outram et al., 2021). Plant proteases can perform an alternative processing of these effectors in the apoplast, disrupting their function and generating elicitors for their own immunity (Wang et al., 2021c). The AVR9 effector expressed by the fungal pathogen *Cladosporium fulvum* can induce HR in tomato plants carrying the resistance gene *Cf9* (Laugé et al., 2000; Kruijt et al., 2005; Wang et al., 2021a). AVR9 is encoded as a pre-proAVR9 (63 aa) comprising an extracellular pro-peptide of 40 aa, which is processed by fungal and plant proteases into a mature protein of 28 aa (De Wit et al., 1985; Van Kan et al., 1991). Extracellular proteases from *C. fulvum* directly processed the 40-aa peptide into intermediate forms of 32, 33, and 34 aa, whereas plant proteases produced a 28-aa peptide (Van den Ackerveken et al., 1993; Vervoort et al., 1997). Interestingly, accumulation of the 28-aa peptide has also been observed in tobacco plants overexpressing pro-AVR9 in the absence of the fungus, indicating that plant proteases can directly process the 40-aa pro-effector into a mature elicitor (Honée et al., 1995). This suggests a struggle between the pathogen and the host to shape a molecule and make it bioactive for their own advantage.

The cysteine-rich protein PC2 is a putative effector secreted into the apoplast as a 208-aa protein during infection of potato by the late blight oomycete *P. infestans* (Wang et al., 2021c). PC2 can be targeted by tomato SBT P69B in the apoplast and subsequently activate BAK1-dependent immune responses, including accumulation of ROS, upregulation of defense-related genes, and cell death (Wang et al., 2021c). The aspartic residue at position 117 in PC2 is essential for precursor processing, but the precise cleavage site is still unknown. Processing of pro-PC2 by host proteases converts the effector to the elicitor.

## Concluding remarks and future perspectives

Activation of immunity is an energy-intensive process that can be detrimental to normal plant growth (He et al., 2022b). Fine tuning of the plant immune system is necessary to mount a strong and effective defense response (Li et al., 2020). Precise on/off mechanisms are necessary for efficient defense activation. Post-translational modifications play a critical role in plant immune responses (Withers and Dong, 2017). These mechanisms include the processing of inactive protein precursors to generate active phytocytokine and DAMP signals in the apoplast for plant immunity. Synergies and interactions between the signaling induced by different immunostimulatory peptides and CW DAMPs have emerged.

Several questions remain unanswered regarding protease/pro-phytocytokine and protease/zymogen pairs, as well as the processing cascades that trigger plant immunity. It is unclear why some precursors are cleaved at the terminal domains and others at the midsection. The role of the pro regions after processing is also largely unknown. Hypothesized functions include targeting the companion enzyme/peptide toward the apoplast, acting as an intramolecular chaperone, or serving as an inhibitor of the mature precursors. Pro regions may also function as additional elicitors. Recently, the pro region of Arabidopsis MC2 was reported to positively regulate PRR-mediated immune signaling by interfering with inhibition of BAK1 by BIR1, a negative regulator of plant immunity (Wu et al., 2023). On the other hand, the structural similarities of the N-terminal pro region of PMEs with functionally characterized PMEIs suggest that they may also act as antimicrobial proteins (An et al., 2008; Coculo and Lionetti 2022). Open questions also include whether each precursor protein has an autonomous role and whether they have additional functions with respect to their processed regions. The possible interplay of the different precursor proteins and the activated immune pathways remains to be elucidated. The maturation of multiple precursor proteins can amplify immunity by activating overlapping pathways. Evidence indicates a cooperation between phytocytokines, CW DAMPs, and remodeling enzymes and other elicitors, but their interplay and the activation dynamics of the different signaling pathways require further investigation. Another point of discussion for future research is whether different secretion routes (conventional and unconventional) are important for organizing substrate processing in the correct subcellular compartment and avoiding premature activation.

A thorough understanding of these molecular mechanisms could have a significant impact on the identification of new eco-friendly strategies for crop protection against a broad spectrum of pathogens. Development of disease control strategies based on direct delivery of CW DAMPs and phytocytokines to plants represents a powerful approach for sustainable agriculture, potentially reducing the use of chemicals while providing food quality and safety. Genome-editing technologies could be used for precise stacking of multiple transgenes encoding mature phytocytokines and CW remodeling enzymes/inhibitors in a single cultivar under the control of a pathogen-inducible promoter. Genetic engineering strategies aimed at overexpressing elicitor receptors or silencing susceptibility factors could also be explored. An environmentally sustainable approach to increasing crop production and health may involve the development of bioformulations capable of modulating plant defense. One example of such a formulation is COS-OGA, the first “low-risk active substance” authorized in Europe containing OGs and chito-oligosaccharides, which can enhance defense responses of grapevine against *Uncinula necator* and potato against *P. infestans* (Van Aubel et al., 2014; Clinckemaillie et al., 2017). Another solution is represented by Messenger, based on Harpin (Ea) protein derived from *E. amylovora*, which enhances disease resistance in a wide variety of economically important crops such as cotton, wheat, cucumber, citrus, tobacco, strawberry, tomato, and peppers against viruses, nematodes, and fungi (Wei and Betz 2006). DAMPs and phytocytokines, either alone or in combination with specific MAMPs, can be used as phytovaccines to improve resistance to pathogens in different plants (Chen et al., 2020; Shen et al., 2022).

## Funding

V.L. is supported by 10.13039/501100004271Sapienza University of Rome, grants RM120172B78CFDF2, RM11916B7A142CF1, RM122181424F1F42, and RG12117A898EABE0, by the 10.13039/501100000780European Union “NextGenerationEU” program “Project ECS 0000024 Rome Technopole” - CUP B83C22002820006, PNRR Missione 4 Componente 2 Investimento 1.5, and by the 10.13039/501100003407Italian Ministry for Education, University and Research (MUR) with the project REACH-XY: CUP B93C22001920001.

## Author contributions

Conceptualization, V.L.; writing – original draft, D.D.C. and V.L.; writing – review & editing, V.L., D.C., D.D.C., M.G., and G.D.L.; funding acquisition, V.L.
